# A comparison of general and ambulance specific stressors: predictors of job satisfaction and health problems in a nationwide one-year follow-up study of Norwegian ambulance personnel

**DOI:** 10.1186/1745-6673-6-10

**Published:** 2011-03-31

**Authors:** Tom Sterud, Erlend Hem, Bjørn Lau, Øivind Ekeberg

**Affiliations:** 1 Department of Behavioural Sciences in Medicine, Institute of Basic Medical Sciences, Faculty of Medicine, University of Oslo, PO Box 1111 Blindern, NO-0317 Oslo, Norway; 2 National Institute of Occupational Health, Oslo, Norway

## Abstract

**Objectives:**

To address the relative importance of general job-related stressors, ambulance specific stressors and individual characteristics in relation to job satisfaction and health complaints (emotional exhaustion, psychological distress and musculoskeletal pain) among ambulance personnel.

**Materials and methods:**

A nationwide prospective questionnaire survey of ambulance personnel in operational duty at two time points (n = 1180 at baseline, T1 and n = 298 at one-year follow up, T2). The questionnaires included the Maslach Burnout Inventory, The Job Satisfaction Scale, Hopkins Symptom Checklist (SCL-10), Job Stress Survey, the Norwegian Ambulance Stress Survey and the Basic Character Inventory.

**Results:**

Overall, 42 out of the possible 56 correlations between job stressors at T1 and job satisfaction and health complaints at T2 were statistically significant. Lower *job satisfaction at T2 *was predicted by frequency of lack of leader support and severity of challenging job tasks. *Emotional exhaustion at T2 *was predicted by neuroticism, frequency of lack of support from leader, time pressure, and physical demands. Adjusted for T1 levels, emotional exhaustion was predicted by neuroticism (beta = 0.15, p < .05) and time pressure (beta = 0.14, p < 0.01). *Psychological distress at T2 *was predicted by neuroticism and lack of co-worker support. Adjusted for T1 levels, psychological distress was predicted by neuroticism (beta = 0.12, p < .05). *Musculoskeletal pain at T2 *was predicted by, higher age, neuroticism, lack of co-worker support and severity of physical demands. Adjusted for T1 levels, musculoskeletal pain was predicted neuroticism, and severity of physical demands (beta = 0.12, p < .05).

**Conclusions:**

Low job satisfaction at T2 was predicted by general work-related stressors, whereas health complaints at T2 were predicted by both general work-related stressors and ambulance specific stressors. The personality variable neuroticism predicted increased complaints across all health outcomes.

## Introduction

Much research on health in the ambulance service has been based on the assumption that such work is inherently stressful [1,2]. Ambulance workers frequently have to take rapid action and provide medical care under life-and-death circumstances in unfamiliar and inconvenient conditions, while being scrutinized by bystanders and relatives [3]. Ambulance personnel also must attend to non-emergency work, such as transporting and providing appropriate care to chronically and terminally ill patients, which imposes different emotional demands and which might be experienced as more emotionally exhausting than more sensational events [4]. Others have claimed that ambulance work may not be inherently stressful, and that the relatively high level of psychological distress is mainly due to generic organizational stressors that are similar across occupations, such as long hours, workload, lack of control, and little support from managers [5].

Previous research on ambulance work reveals several difficulties in stating firm conclusions about the relative importance of patient care and operational factors compared to sources other than ambulance work, such as the 'managerial role', the 'relations with others at work' and 'general job demands'. Firstly, research concerning both administrative-organizational and ambulance-specific stressors is sparse. Secondly, a potentially important aspect, which has been given little attention, is the distinction between frequency and severity of events. Most studies have considered only the degree of exposure to a stressor [6], without taking into consideration that some situations in ambulance work, such as 'incident with seriously injured children' or 'handling seriously injured persons', may be experienced as very severe stressors that may predispose ambulance personnel to distress and post-traumatic stress symptoms. In comparison, administrative-organizational stressors may be experienced as more frequent and chronic stressors. The most common factors reported to be associated with mental distress among health personnel are work demands (long hours, workload, and pressure), lack of control over work, and poor support from managers [7]. Furthermore, administrative-organizational stressors may not be an expected part of ambulance work and a high frequency level may over time be an important source of frustration and low job satisfaction among ambulance personnel.

Some authors have suggested that individual characteristics might explain the high level of distress symptoms among ambulance personnel [8-10]. In general, factors within the workplace interact with those within the individual to produce levels of fit between people and their jobs, which may lead to greater or less stress. Personality has been postulated to influence stress levels, partly through having an effect on the frequency of exposure to stressors, but more importantly, through modifying the experience of stress severity associated with the stressors [11]. We therefore decided to explore the possibility that personality influences distress levels among ambulance personnel, and at the same time consider the possibility that the relationship between job stressors and health outcomes is spurious because certain personality traits may cause some people to be vulnerable both to job-related stress and health complaints. Moreover, being female in a male-dominated working environment such as the ambulance services may be a risk factor for higher levels of job stress among ambulance women. Older employees, on the other hand, are more experienced and may therefore experience potentially traumatic stressors as less severe, but may nevertheless be more vulnerable to physical demands and musculoskeletal pain.

Based on this background information, we studied the relative importance of general job-related stressors, ambulance specific stressors, and individual characteristics in a one-year follow-up study of Norwegian ambulance personnel. The longitudinal design allowed that the independent and dependent variables were measured at different times.

We wanted to address the following hypotheses:

• Ambulance work is inherently stressful and health complaints among ambulance personnel are mainly related to ambulance specific stressors.

• Health complaints and low job satisfaction among ambulance personnel are mainly related to general job-related stressors.

• Differences in psychological distress among ambulance personnel are mainly related to individual characteristics (personality, age and gender).

## Materials and methods

### Procedure

In April 2005, questionnaires were distributed to the ambulance chiefs in all 19 ambulance regions in Norway. They had agreed to distribute the questionnaire to all ambulance personnel in the ambulance stations within their regions. This procedure was chosen because, at the time, no central national register covering all employed ambulance personnel in Norway was available. Two written reminders were distributed through the ambulance chiefs, and the two major worker union organizations encouraged their members to answer the questionnaire in their homepages and their membership journals. In total, 3200 questionnaires were distributed. Based on reports from four of the ambulance chiefs, 64 ambulance personnel were excluded because they were no longer in service. In total, 1286 persons returned questionnaires (41%). Unfortunately, we were not able to get fully updated address lists from the other ambulance chiefs. Hence, it is likely that questionnaires were distributed to persons who were no longer active ambulance personnel, or were on leave, in these regions. Thus, the real response rate is most likely higher than 41%. Analysis of variance was used to compare mean levels on the included variables and to test the assumption that the bivariate associations were similar in those who responded in the main round and those who responded after one or two reminders. We found no significant differences in mean scores, and no significant interactions between the bivariate associations and time of response.

**Figure 1:**
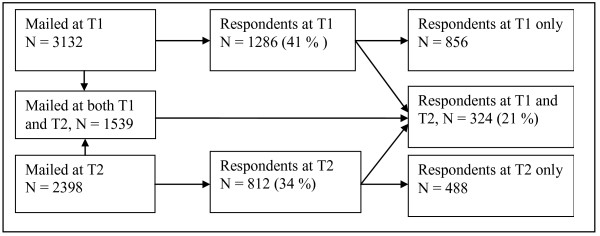
**Description of the ambulance sample**.

Because of the problems in the first distribution rounds, we decided to take advantage of the address lists obtained from The Norwegian Registration Authority for Health Personnel (SAFH) in the one-year follow-up. In May 2006, a shortened questionnaire--12 pages compared with 20 pages at T1--was distributed to the registered home addresses of 2,398 persons who were registered as authorized or licensed ambulance personnel. One follow-up reminder was distributed. Figure 1 provides a description of the sampling procedure. In total, 812 persons returned their questionnaire (34%). Out of these, 324 responded also at T1. Due to the lack of overlap between the two address lists only 1539 persons received the questionnaire at both time-points. The response rate among respondents at both time points was estimated to 21 percent (324/1539).

### Sample

Participants in this study included officers, middle managers and managers who reported to do ambulance work more than 50% of their work-time (N = 1180 at T1). The term 'operational ambulance personnel' is used to describe these respondents. Among the operational ambulance personnel who responded at T1 644 persons received the questionnaire at T2. Thus the response among these respondents was estimated to 50 percent at T2 (324/644). Among the respondents at T1, 76.8% were men. The age of the participants ranged from 18 to 66, with a mean age of 36.8 (SD = 9.3); the mean age was 37.6 (9.0) for men and 33.8 (9.6) for women (*p *< .001). The subsample who responded at both T1 and at T2 (one-year follow up) (N = 324) were significantly older and had a higher proportion of male personnel compared to respondents at T1. Overall, however, there were small differences between respondents at both T1 and T2 compared to the sample who answered at T1 (see Table 1). In order to take advantage of the prospective design, regression analyses was performed using this sample (n = 298 after listwise deletion).

**Table 1:** Means, standard deviations and comparison between the sample available at T1 only and the sample available at both T1 and T2.

	Sample 1		Sample 2		T-test
	(T1 only)		(T1 and T2)		sample 1 vs. sample 2
	(n = 784-821)		(n = 310-324)		
**Dependent variables measured at T1 and T2**	**Mean**	**SD**	**Mean**	**SD**	
Job satisfaction at T1(1-9)	3.1	0.5	3.2	1.0	
Job satisfaction at T2			3.2	-0.9	
Emotional exhaustion at T1 (1-5)	2.0	0.6	2.0	-0.6	
Emotional exhaustion at T2			2.0	0.7	
Psychological distress at T1(0-4)	0.4	0.5	0.4	0.5	
Psychological distress T2			0.3	0.5	
Musculoskeletal pain T1 (0-21)	3.4	3.2	3.3	2.9	
Musculoskeletal pain T2			3.5	3.0	
					
Independent variables measured at T1					
Women (%)	25.6		16.9		**
Age (18-60)	36.3	9.4	38.2	8.9	**
Neuroticism (0-9)	2.8	2.2	2.6	2.2	
Control (0-9)	3.6	2.2	3.9	2.0	
Exstroversion (0-9)	5.6	2.3	5.5	2.3	
Self-efficacy (1-5)	3.0	0,5	3.1	0,5	*
Lack of co-worker support (F) (0-9)	3.1	2.8	3.5	3.0	*
Lack of leader support (F) (0-9)	1.9	2.2	2.2	2.4	*
Time pressure frequency (F) (0-9)	2.0	2.1	2.2	2.2	
Challenging job tasks (F) (0-9)	2.6	1.9	2.7	1.9	
Lack of co-worker support (S) (1-9)	5.3	1.7	5.5	1.6	
Lack of leader support (S) (1-9)	5.1	1.7	5.1	1.7	
Time pressure (S) (1-9)	4.3	1.5	4.3	1.4	
Challenging job tasks (S) (1-9)	4.4	1.3	4.3	1.3	
Non-emergency tasks (F) (0-9)	2.8	2.0	2.9	2.1	
Physical demands (F) (0-9)	5.6	3.3	5.9	3.2	
Serious Operational tasks (F) (0-9)	2.8	2.0	3.0	2.0	
Non-emergency tasks (S) (1-9)	4.5	1.4	4.3	1.5	*
Physical demands (S) (1-9)	5.4	1.8	5.3	1.9	
Serious Operational tasks (S) (1-9)	5.8	1.4	5.7	1.5	

Note. A series of t-tests was conducted and there were no significant differences among those who answered at T1 only to respondent who answered at both T1 and T2. A series of paired sampled t-tests was conducted, and there were no significant changes from T1 to T2 for each of the outcome measures.

#### Dependent variables

Emotional exhaustion was measured with nine items from the Maslach Burnout Inventory--Human Services Survey [12]. The items are scored on a five-point scale ranging from 1 to 5 during the last 14 days. The score was computed as the mean of valid responses (α = 0.86).

The Job Satisfaction Scale consists of ten questions examining various aspects of working conditions and stressors: responsibility, variation, collaboration, salary, working hours, etc. (α = 0.85) [13]. All items were scored on a scale from 1 (extremely satisfied) to 7 (extremely dissatisfied). The score was computed as the mean of valid responses.

Psychological distress was measured by SCL-10, a 10 items version of the Symptom Check List-25 [14]. The shorter versions of SCL-10 has been reported to perform almost as well as the full version [15]. Each item was measured on a five-point scale from not at all (0) to very much (4). The score was computed as the mean of valid responses (α = 0.88).

Musculoskeletal pain was assessed by 7-items from the Subjective Health Complaint questionnaire [16]. The items (i.e. shoulder, upper back, low back, neck, arm, leg pain during physical activity and headache/migraine) are scored on a four-point rating scale ranging from no complaints (0) to serious complaints (3). Each complaint is also scored for duration (number of days) during the last 30 days, but this information was not considered in the present analysis. The score was computed as the mean of valid responses (α = 0.7).

#### Severity and Frequency of general stressors

General organizational stressors was measured with the Job Stress Survey (JSS) [17]. The instrument describe 30 stressors that are rated on a nine-point perceived severity and frequency rating scale from 0 to 9+, in relation to the last six months We performed a principal component analysis with varimax rotation. All items were measured at T1 only, and the analysis was performed on the total T1 sample. The analysis resolved as four factors (62 percent cumulative explained variance, based on 19 items): 'time pressure' (five items α = .82), 'challenging job tasks' (five items, α .78), 'lack of leader support' (six items, α = .88), and 'lack of co-worker support' (three items, α = .78). A similar factor structure was also supported for the frequency items. The instrument is described in greater details elsewhere [18]

#### Ambulance specific stressors

The Norwegian Ambulance Stress Survey (NASS) was constructed especially for the present study to measure ambulance-specific stressors. The instrument consists of 29 items that are described and assessed in the same way as the Job Stress Survey. To identify a factor we performed a principal component analysis with varimax rotation. All items were measured at T1 only, and the analyses were performed on the total T1 sample. The analysis resolved as three factors (65 percent cumulative explained variance, based on 14 items,), with good conceptual meaning: 'non-emergency tasks' (five items, α = .80), 'serious operational tasks' (six items, α = .85), and 'physical demands' (three items, α = .93). A similar factor structure was also supported for the frequency items. The instrument is described in greater details elsewhere [18]

#### Individual characteristics

Personality was measured by 27 items from the Basic Character Inventory (BCI) [19]. BCI is based on the 'big three' personality dimensions: Neuroticism (α = .74), Extroversion (α = .72) and Control (α = .66). Each dimension is based on nine questions with a dichotomous response (0 = does not apply, 1 = applies), allowing each dimension a range of scores between 0 (low) and 9 (high).

Gender was coded with women as a reference category. Age was treated as a continuous variable.

### Statistics

SPSS version 17.0 was used for statistical analyses. Means and frequencies were used to describe the data in the present study, and t-tests and chi-square tests were used to test for differences across samples. Principal component analysis with varimax rotation was used to check the factor structure of the instruments. Simultaneous effects of the included independent variables were estimated by multiple linear regression analyses (OLS). A stepwise procedure was chosen in order to identify individual characteristics and job-related stressors that significantly predicted job satisfaction and health complaints at follow-up. In models with even number (2, 4, 6, 8) all analyses were adjusted for T1 levels on the relevant dependent variable. From the multiple regression analyses, both standardized beta values and squared semi-partial correlation coefficients (e.g. part correlation in the SPSS output) are reported. The squared semi-partial correlations provide a means of assessing the relative "importance" of the independent variables in determining Y, and shows how much each variable uniquely contributes to R2 over and above that which can be accounted for by the other predictors.

## Results

Table 1 provides the means and standard deviations (median and range for categorical variables) for the study variables for respondents at T1 only (sample 1) and respondents at both T1 and T2 (sample 2). Sample 2 was significantly older (38.2 vs. 36.7, p < .01) and had a significantly higher proportion of male respondents (84% vs. 77%, p < .01) compared to the sample at T1. A series of t-tests were conducted and there were no significant differences between sample 1 and sample 2 on any of the outcome variables. Respondents in sample 2 had a significantly higher score on two and a significantly lower score on one out of the total of fourteen job stressors.

Table 2 provides Pearson's correlations between the dependent variables measured at T1 and T2 and the independent variables measured at T1. Overall, 42 out of the possible 56 correlations between job stressors at T1 and the dependent variables at T2 were statistically significant. Severity of serious operational demands was the only stressor not significantly related to any of the outcome variables at T2. Neuroticism was significantly related to all health outcomes at T2. Gender and age differences were found for musculoskeletal pain.

**Table 2:** Bivariate Pearson's correlations between independent variables measured at T1 and job satisfaction, emotional exhaustion, psychological distress and musculoskeletal pain measured at T1 and T2 (sample 3, n = 298 after listwise deletion).

		1	2	3	4	5	6	7	8	9	10	11	12	13	15	16	17	18	19	20	21	22	23	24	25	26	27
1	Low job satisfaction at T1																										
2	Low job satisfaction at T2	**.60**																									
3	Emotional exhaustion at T1	**.50**	**.36**																								
4	Emotional exhaustion at T2	**.32**	**.50**	**.63**																							
5	Psychological distress at T1	**.30**	**.18**	**.58**	**.39**																						
6	Psychological distress T2	**.15**	**.30**	**.51**	**.67**	**.64**																					
7	Musculoskeletal pain T1	**.13**	.08	**.41**	**.29**	**.41**	**.40**																				
8	Musculoskeletal pain T2	.11	**.14**	**.28**	**.35**	**.34**	**.42**	**.61**																			
9	Gender (women = 0. men = 1)	**.12**	.05	.07	.02	-.05	-.04	**-.12**	**-.12**																		
10	Age	-.03	-.07	-.08	-.04	-.01	-.02	.11	**.13**	**.15**																	
11	Neuroticism	**.18**	.09	**.39**	**.34**	**.56**	**.44**	**.25**	**.24**	-.08	-.01																
12	Control	-.01	.01	.09	.09	**.18**	**.15**	.00	.05	-.11	.07	**.19**															
13	Ekstroversion	.08	.11	-.05	-.03	**-.14**	-.07	-.06	.00	-.11	-.10	**-.30**	-.07														
14	Lack of co-worker support (F)	**.29**	**.24**	**.36**	**.27**	**.25**	**.21**	.09	**.14**	-.02	**-.29**	**.16**	.05	**.15**													
15	Lack of leader support (F)	**.49**	**.36**	**.43**	**.24**	**.27**	**.17**	**.12**	**.13**	-.01	**-.25**	**.12**	.01	**.13**	**.55**												
16	Time pressure frequency (F)	**.22**	**.15**	**.35**	**.27**	**.21**	**.14**	.04	.10	.08	**-.16**	**.15**	.06	.06	**.60**	**.42**											
17	Challenging job tasks (F)	**.21**	.10	**.33**	**.19**	**.14**	.07	.04	.07	.10	**-.22**	.10	-.10	.08	**.59**	**.51**	**.64**										
18	Lack of co-worker support (S)	**.30**	**.20**	**.28**	**.26**	**.25**	**.23**	**.12**	.11	.02	**-.17**	**.21**	.00	.00	**.48**	**.26**	**.24**	**.20**									
19	Lack of leader support (S)	**.47**	**.34**	**.38**	**.24**	**.30**	**.15**	**.16**	.10	.01	**-.16**	**.17**	-.04	.00	**.30**	**.58**	**.22**	**.21**	**.50**								
20	Time pressure severity (S)	**.22**	**.18**	**.29**	**.27**	**.17**	.11	**.18**	.09	**.12**	.09	**.17**	-.07	-.05	.08	-.01	**.21**	.03	**.51**	**.36**							
21	Challenging job tasks (S)	**.37**	**.30**	**.27**	**.26**	**.19**	**.15**	**.13**	.09	.09	.02	**.26**	.03	**-.15**	.01	**.12**	.05	.01	**.41**	**.51**	**.56**						
22	Non-emergency tasks index (F)	**.17**	.09	**.32**	**.25**	**.15**	**.13**	-.04	.04	.02	**-.24**	**.13**	.02	.07	**.60**	**.44**	**.63**	**.61**	**.25**	**.19**	.05	.01					
23	Physical demands (F)	**.17**	**.12**	**.26**	**.21**	.09	.09	.08	**.12**	.03	**-.12**	.03	-.04	.06	**.46**	**.35**	**.53**	**.50**	**.19**	**.19**	.10	.06	**.55**				
24	Serious Operational tasks (F)	.12	.08	**.22**	**.16**	.07	.07	.01	.06	.04	**-.20**	.06	-.05	.08	**.48**	**.40**	**.57**	**.58**	**.13**	**.12**	-.02	-.02	**.71**	**.65**			
25	Non-emergency tasks index (S)	**.20**	**.16**	**.25**	**.22**	**.20**	**.12**	**.12**	**.13**	.03	-.05	**.23**	-.04	-.08	.05	.00	.08	-.02	**.45**	**.36**	**.66**	**.59**	.06	.05	-.02		
26	Physical demands (S)	**.24**	**.20**	**.34**	**.27**	**.23**	**.21**	**.27**	**.27**	-.07	.11	**.18**	.01	-.04	.08	.07	.11	.06	**.24**	**.16**	**.39**	**.33**	.06	**.23**	.06	**.46**	
27	Serious Operational tasks (S)	**.13**	**.07**	**.20**	.09	**.12**	.06	.11	.07	-.09	.04	**.21**	.00	-.11	-.05	-.04	.03	-.06	**.32**	**.26**	**.43**	**.44**	.02	.01	-.03	**.67**	**.45**

Note. All tests two-tailed; Pearson's r significant at p < .05 marked in bold; S = Severity level and F = Frequency level

Table 3 presents the results from the multiple linear regression analyses. Low job satisfaction at T2 was predicted by frequency of lack of leader support and severity of challenging job tasks. After adjusting for job satisfaction at T1 (beta = 0.59, p < .01), there were no significant predictors of job satisfaction at T2.

**Table 3:** Multiple regressions on job satisfaction, emotional exhaustion, psychological distress and musculoskeletal pain measured at T2, unadjusted and adjusted for T1 levels (sample 3, n = 298 after listwise deletion).

	Job satisfaction at T2		Emotional exhaustion at T2		Psychological distress at T2		Musculoskeletal pain at T2	
	model#1	model#2	model#3	model#4	model#5	model#6	model#7	model#8
	beta (R2)	beta (R2)	beta (R2)	beta (R2)	beta (R2)	beta (R2)	beta (R2)	beta (R2)
*Adjusting for T1 levels*		0.59***(0.35)		0.59***(0.28)		0.59***(0.25)		0.57***(0.29)
*Individual charachteristics*								
Men							-0.14*(0.02)	
Age							0.17**(0.03)	
Neuroticism			0.31*** (0.09)	0.15** (0.02)	0.41***(0.17)	0.12*(0.01)	0.19***(0.03)	0.11*(0.01)
Control								
Ekstroversion	0.12*(0.01)							
*General stressors*								
Lack of co-worker support (F)							0.16*(0.02)	
Lack of leader support (F)	0.32***(0.10)		0.13* (0.01)					
Time pressure frequency (F)			0.12* (0.01)					
Challenging job tasks (F)								
Lack of co-worker support (S)					0.15**(0.02)			
Lack of leader support (S)								
Time pressure severity (S)			0.19*** (0.03)	0.14** (0.02)				
Challenging job tasks (S)	0.27**(0.07)							
*Ambulance specific stressors*								
Non-emergency tasks index (F)								
Physical demands (F)								
Serious Operational tasks (F)								
Non-emergency tasks index (S)								
Physical demands (S)			0.17** (0.02)				0.22***(0.05)	0.12*(0.01)
Serious Operational tasks (S)			-0.13* (0.01)	-0.12* (0.01)				

Adjusted R2 for the final models	0.195	0.351	0.245	0.464	0.212	0.348	0.163	0.397

*P < 0.05; **P < 0.01, ***P < 0.001; beta = standardized beta coefficients; a one unit change in age represents 10 years; (R2) = squared semi-partial correlation; F = frequency and S = severity.

Emotional exhaustion at T2 was predicted by neuroticism, frequency of lack of leader support, severity and frequency of time pressure, severity of physical demands and severity of operational demands. After adjusting for emotional exhaustion at T1 ((beta = 0.59, p < .01), emotional exhaustion at T2 was predicted by neuroticism (beta = 0.15, p < .05), severity of time pressure (beta = 0.14, p < .01) and severity of operational demands (beta = -0.12, p < .05).

Psychological distress at T2 was predicted by neuroticism and severity of lack of co-worker support. After adjusting for psychological distress at T1 (beta = 0.59, p < .01), psychological distress at T2 was predicted by neuroticism (beta = 0.12, p < .05).

Musculoskeletal pain at T2 was predicted by being female, older age, neuroticism, frequency of lack of co-worker support and severity of physical demands. After adjusting for musculoskeletal pain at T1 (beta = 0.56, p < .01), musculoskeletal pain at T2 was predicted by neuroticism (beta = 0.11, p < .05) and severity of physical demands (beta = 0.12, p < .05).

## Discussion

This study showed that health symptoms at one-year follow-up were predicted by both general stressors and ambulance specific stressors at baseline. However, after adjusting for initial level of health complaints, there were few significant predictors of increased health complaints at follow-up. This stability could be explained by the fact that the sample is a rather homogeneous group and their job conditions stay rather equal, at least in a one-year perspective. For example, if lack of support from leaders over a significant time has reduced job satisfaction both at T1 and at T2, the T1 level will most likely explain most of the variance at T2 if the situation is rather stable. The data does not, however, allow us to test the direction of the relationship between self-reported exposure levels at T1 and initial levels of poor health and job satisfaction. Initial poor health may be considered a confounder to the extent that it has an effect on the reporting of exposure levels at T1, but it may also be considered a mediator if it is a consequence of previous exposure.

Low job satisfaction at T2 was most strongly related to general occupational stressors. A relatively high level of job satisfaction has been reported in earlier studies among ambulance personnel [20,21]. However, a distinction between satisfaction with regard to the job and satisfaction with regard to the organization can be made. In contrast to the critical incidents and more routine emergency calls, ambulance personnel must alternatively cope with the boredom and tedium associated with waiting for the next alarm. This time at the station can foment administrative and also co-worker tension and conflicts, which accords with the finding that frequency of lack of leader support and severity of challenging job tasks predicted lower job satisfaction at T2.

An important finding was that, although the ambulance specific stressor serious operational tasks has been shown to be ranged as the most severe stressor [18], it was not related to health problems at T2. In fact, the adjusted estimates of serious operational tasks were negatively related to emotional exhaustion and psychological distress at T2. A possible interpretation of these findings is that although ambulance personnel have to deal with a diversity of ambulance specific incidents that are ranged as severe, these types of stressors are most likely an expected part of their occupation, and therefore most ambulance workers may be able to cope with these events reasonably well.

Frequency of lack of leader support was found to predict emotional exhaustion and low job satisfaction at T2, and severity of lack of co-workers support was found to predict psychological distress at T2. These results concur with other studies that have reported that social aspects of the work environment predict higher levels of psychological distress and emotional exhaustion among ambulance personnel [22,23]. However, in the present study, the severity of time pressure was the only job stressor to predict an increase in job-related emotional exhaustion from T1 to T2.

Severity of physical demands was found to predict higher levels of emotional exhaustion and musculoskeletal pain at T2, and importantly, was found to predict an increase in musculoskeletal pain from T1 to T2. Other studies have reported that ambulance personnel report higher levels of physical strain than employees in other health services [23], and that ambulance personnel self-report more musculoskeletal and physical health problems than the general population [24,25]. This study further shows that heavy lifting and carrying under difficult conditions is an important stressor to consider in the ambulance occupation.

The personality trait neuroticism was the most important predictor of psychological distress, and was also found to predict an increase in emotional exhaustion and musculoskeletal pain from T1 to T2. A characteristic in highly neurotic is that they are continually preoccupied with their inadequacies. They are likely to show depressive affect as a consequence of contemplating their shortcomings because they find so little that is tolerable within. On the other hand, however, it is noteworthy that personality was marginally related to low job satisfaction, which may indicate that the ambulance services successfully attract a type of people who are highly motivated to do this kind of work.

Being female in a male-dominated working environment such as the ambulance services does not seem to be a risk factor for mental health problems among ambulance women. Moreover, there were no age differences in mental health problems. However, higher age predicted higher levels of musculoskeletal pain at one-year follow-up. A higher level of musculoskeletal pain among the older workers is in accordance with what has been found in other studies [26].

### Strengths and limitations

The strengths of this study are that it is one of the largest investigations of ambulance personnel conducted, it is nationwide, and has a longitudinal design. The response rate was moderate, which may question the representativeness of the data. This is an important issue, because people who refuse to participate may have more health problems. However, there was no difference in the mean levels on the stress indicators between those who returned the questionnaire early, and those who returned it late at T1. As late responders may resemble the non-respondents [27], the lack of representativeness may not be a severe problem. Further, because of the problems in the questionnaire distribution, it is likely that the real response rate is higher than the estimated proportion. Overall, there were small and non substantial differences between respondents at both T1 and T2 compared to the sample who answered at T1. Thus, the sample who answered at both time points was found to be reasonably representative for the total sample at T1.

## Conclusions

Low job satisfaction at one-year follow-up was predicted by the general stressors lack of leader support and challenging job tasks, whereas health complaints at one-year follow-up were predicted by both general stressors and ambulance specific stressors.

Lack of support from leaders and co-workers predicted higher levels of burnout and psychological distress at one-year follow-up, whereas ambulance specific physical demands predicted higher levels of emotional exhaustion and musculoskeletal pain at one-year follow-up. The personality variable neuroticism was an independent predictor of an increase across all health complaints over the one-year follow-up period. Even if ambulance personnel will have problems if they are too vulnerable, moderate levels of neuroticism is common. Both colleagues and leaders should be aware of that, and possibly be more supportive and encouraging in an occupation that has had a reputation of being too masculine.

## Competing interests

The authors declare that they have no competing interests.

## Authors' contributions

TS, EH, BL and ØE were involved in the conception and design of the study, interpretation of data and critical revisions of the manuscript. TS performed the statistical analyses and drafted the manuscript. TS will act as guarantor for the paper. All authors approved the final manuscript.
